# Inferring the three-dimensional structures of the X-chromosome during X-inactivation

**DOI:** 10.3934/mbe.2019369

**Published:** 2019-08-12

**Authors:** Hao Zhu, Nan Wang, Jonathan Z. Sun, Ras B. Pandey, Zheng Wang

**Affiliations:** 1 Department of Computer Science, University of Miami, 1364 Memorial Drive, Coral Gables, FL 33124, USA; 2 Department of Computer Science, New Jersey City University, 2039 Kennedy Blvd, Jersey City, NJ 07305, USA; 3 Department of Computer Science, College of Charleston, Charleston, SC 29424, USA; 4 Department of Physics and Astronomy, University of Southern Mississippi, 118 College Drive #5046, Hattiesburg, MS 39406, USA

**Keywords:** 3D genome, 3D cubic lattice, simulation, Xist, lncRNA, X-chromosome inactivation

## Abstract

The Hi-C experiment can capture the genome-wide spatial proximities of the DNA, based on which it is possible to computationally reconstruct the three-dimensional (3D) structures of chromosomes. The transcripts of the long non-coding RNA (lncRNA) Xist spread throughout the entire X-chromosome and alter the 3D structure of the X-chromosome, which also inactivates one copy of the two X-chromosomes in a cell. The Hi-C experiments are expensive and time-consuming to conduct, but the Hi-C data of the active and inactive X-chromosomes are available. However, the Hi-C data of the X-chromosome during the process of X-chromosome inactivation (XCI) are not available. Therefore, the 3D structure of the X-chromosome during the process of X-chromosome inactivation (XCI) remains to be unknown. We have developed a new approach to reconstruct the 3D structure of the X-chromosome during XCI, in which the chain of DNA beads representing a chromosome is stored and simulated inside a 3D cubic lattice. A 2D Gaussian function is used to model the zero values in the 2D Hi-C contact matrices. By applying simulated annealing and Metropolis-Hastings simulations, we first generated the 3D structures of the X-chromosome before and after XCI. Then, we used Xist localization intensities on the X-chromosome (RAP data) to model the traveling speeds or acceleration between all bead pairs during the process of XCI. The 3D structures of the X-chromosome at 3 hours, 6 hours, and 24 hours after the start of the Xist expression, which initiates the XCI process, have been reconstructed. The source code and the reconstructed 3D structures of the X-chromosome can be downloaded from http://dna.cs.miami.edu/3D-XCI/.

## 1. Introduction

Chromosome conformation capture (3C) [1] is a method that can be used to obtain the spatial organization of the DNA. Its data reflect the interaction frequencies between a single pair of loci on the genome (“one vs one”). The 3C-on-chip (4C) [2] technology was developed to map the interactions between a locus and all other regions (“one vs all”). The 3C-Carbon Copy (5C) [3] method was invented to detect the interactions between multiple pairs of genomic loci (“many vs many”). The interactions within the whole genome can be detected by the Hi-C technique [4], a method that combines proximity-based ligation with massively parallel sequencing to probe the three-dimensional proximation relationships within the genome (“all vs all”). Population Hi-C method is based on a population of cells and has been used in many studies, such as modeling the structures for balanced and unbalanced chromosomal rearrangements in primary human tumor samples [5], detecting the unknown 3D organization of chromosomes during human brain development [6], discovering that the 3D chromatin landscape is relatively stable once established in a particular cell type [7], and demonstrating that individual chromosomes maintain domain organization at the megabase scale [8]. Moreover, single-cell Hi-C method has been developed to reveal cell-to-cell variabilities in terms of chromosome structures [9]. Although we have developed computational approach [10] to reconstruct chromosomal 3D structures based on single-cell Hi-C data, this study will focus on using population Hi-C data due to the availability limitation of single-cell Hi-C data for X-chromosome inactivation.

The 3C, 4C, 5C, and population Hi-C data have been widely used to reconstruct the 3D structures of chromosomes by many computational methods. Duan *etal.* [11] constructed a 4C-based model that provided a way to observe the structure and function of a eukaryotic genome. Bau *et al.* [12] generated high-resolution 3D models of chromatins at megabase scale by developing an approach that combined 5C data with the Integrated Modeling Platform (IMP). Tanizawa *et al.* [13] explored the model organism fission yeast by utilizing an approach combining the 3C data and high throughput sequencing. The above-mentioned methods use multidimensional scaling (MDS), which model the 3D structures of chromosomes by making the Euclidean distance between each pair of beads as close as possible to the target distance that is converted from the number of 3C, 4C, or 5C contacts.

Zhang *et al.* [14] built the ChromeSDE method that improves the MDS objective function. Ben-Elazar *et al.* [15] proposed a statistical framework to reconstruct chromatin structures using a minimum set of assumptions. PASTIS [16] assumes the Hi-C contact counts following a Poisson distribution; and these contact counts decrease with the physical distances between genomic loci. Combing the Poisson counts with target distance, PASTIS was used to generate enzymes structures in different environments. Hu *et al.* [17] developed Bayesian probabilistic approaches BACH and BACH-MIX to study the structural variation of chromatin in a cell population using high resolution Hi-C dataset. Zou *et al.* [18] developed HSA, which globally searches the latent structure underlying different cleavage footprints; its robustness and accuracy outperform existing tools. Oluwadare *et al.* [19] developed a maximum likelihood algorithm called 3DMAX, which automatically re-estimates the conversion factor for converting interaction frequency to target distance and is more robust to structural variability and noise.

Dieter W. Heermann *et al.* [20] built a computational model for simulating the 3D structure of the chromosome fiber on a coarse-grained level. Depending on the high complexity for establishing large-scale chromosomes, they systematically eliminated smaller details such as nucleosomes or small-scale chromatin loops to obtain an all-scale compact globular structure rather than the small-scale linear structures. They accomplished the modeling by using coarse-grained lattice Monte Carlo [21] that puts the chromosomes chain into a large cubic and randomly selected a monomer to move to the neighboring positions.

The X-linked genes in female mammal cells are twice of those in male cells because females have two X-chromosomes, whereas males only have one. The dose of double X-linked genes may pose a great harmful influence to the individual; and mammalian females have evolved a unique way called X-chromosome inactivation (XCI) to eliminate the gene imbalance [22]. The XCI does not delete the extra X-chromosome directly but rather, spreads Xist RNA transcripts on one of two X-chromosomes to silence the redundant gene expressions [23–26]. After XCI, one X-chromosome is compressed into a compact structure and silenced in terms of gene expression in the regular condition [27].

It is important to detect the 3D structure of the inactive X-chromosome during the XCI process. Many studies are based on the structure of the active and inactive X-chromosome to study the mechanism of the X-chromosome inactivation. For example, Bonora and Disteche [28] used traditional microscopy and super-resolution technology to reveal uneven compaction of the inactive X-chromosome. Naughton *et al.* [29] presented a high-resolution chromatin fiber analysis of transcriptionally active and inactive X and showed that the formation of facultative heterochromatin depends on factors at a level above the 30 nm fiber and transcription does not alter bulk chromatin fiber structures. By isolating a comprehensive protein interactome for Xist RNA [30], the study of Minajigi *et al.* unveiled many layers of inactive X-chromosome repression and demonstrated a central role for Xist RNA in the topological organization of mammalian chromosomes. Nora *et al.* [31] used 5C and super-resolution microscopy to analyze the spatial organization of a 4.5 Mb region including Xist RNA on active and inactive X-chromosome. They uncovered a series of discrete 200 kb–1 Mb topologically associating domains (TADs) and the disruption of a TAD boundary causing ectopic chromosomal contacts and long-range transcriptional mis-regulation. In the study of [32], Deng *et al.* applied a recently developed Hi-C assay to mouse F1 hybrid systems and discovered a specific bipartite organization of the mouse inactive X-chromosome that may reveal the maintenance of gene silencing. Chaumeil *et al.* [33] concluded that the chromatin and genome structure influenced the epigenetic control of XCI.

In mammal genomes, Xist, a long non-coding RNA (lncRNA) in the X-chromosome, can localize on the X-chromosome and silence gene expressions by condensing the 3D structure of the X-chromosome. Engreitz *et al.* [34] triggered the XCI process by using tetracycline on male mouse ES cells to start the expressions of the Xist transcripts. They detected the Xist localization intensities on the entire X-chromosome at five time points: 0 hours, 3 hours, 6 hours, 24 hours, and 48 hours after tetracycline was applied by using a biochemistry experiment named RNA Antisense Purification (RAP) [34]. They have found that Xist transcripts spread to spatially proximal sites and uses its a-repeat domain to spread over the active genes when encounters a new region. A-repeat domain may allow Xist to recruit PRC2 or other proteins to modify and compact chromatin that reposition nearby regions into the Xist RNA compartment. Through this process, Xist compartment will grow that pull the interacted region closer to the Xist transcription locus to propagate Xist spreading [34]. They have discovered that the regions with higher localization intensity of Xist usually have shorter spatial distances with the Xist locus (a significant correlation between the number of Hi-C contacts and Xist localization intensities). Recent conformation capture approaches [35] have generated the Hi-C data of the X-chromosome 0 hours and 48 hours after doxycycline induction (the start and end of the XCI process) that are associated with the active and inactive X-chromosomes, respectively. However, the Hi-C data of the X-chromosome during the process of XCI are still not available so that the 3D structures of the X-chromosome during XCI are still unknown.

In this study, we represented a chromosome as beads-on-a-string. Unlike other existing methods, we put it into a 3D cubic lattice and then conducted Metropolis-Hasting simulations. Based on the Xist RAP data, we then inferred the 3D structures of the X-chromosome at 0 hours, 3 hours, 6 hours, 24 hours, and 48 hours after the start of XCI.

## 2. Materials and method

### 2.1. Hi-C data

The Hi-C data of Cast (Xa, or active X-chromosome, treated as the 0 hours X-chromosome in this research) and 129s (Xi, or inactive X-chromosome, treated as the 48-hour X-chromosome after XCI) of mouse neural progenitor cells (NPC) were downloaded from Gene Expression Omnibus (GEO, https://www.ncbi.nlm.nih.gov/geo/query/acc.cgi?acc=GSE72697) with ID GSM1868576. From the same GEO entry but with IDs GSM1868576 and GSM2053973, we downloaded the Hi-C data of mouse embryonic stem cells before and after the doxycycline induction, which were used as the data before and after XCI in our research.

### 2.2. Xist localization data

The Xist RAP data from GEO IDs GSM1141197 to GSM1141201 are the Xist RNA-seq data at 0 hours, 3 hours, 6 hours, 24 hours, and 48 hours after the induction of doxycycline on mouse embryonic stem cells. We downloaded these data from GEO (https://www.ncbi.nlm.nih.gov/geo/query/acc.cgi?acc=GSE46918) that were published in the study of Engreitz *et al.* [34], which provides RAP data for all chromosomes. We only used the RAP data on X-chromosome for our research.

### 2.3 Lattice-based approach to reconstruct chromosome 3D structures

A chromosome is represented as a chain of DNA beads. Each bead is the size of the resolution, e.g., 1 Mb, 500 kb, 250 kb, and 40 kb. For initialization, the program puts the first bead randomly into the cubic lattice. When putting the n-th bead (n>=2), the n-th bead must have a distance of (2, $\sqrt{10}$) with the (n-1)-th bead [36]. Also, to prevent knotting, the n-th bead cannot be smaller than 2 or equal $\sqrt{8}$ to all previously existent beads in the cubic lattice-based on the protocol in [36]. The restriction setting between (2, $\sqrt{10}$) with an exception of $\sqrt{8}$ are excluded volume constraints and the limitations on the changes in the covalent bond length, which have been used in simulating polymer molecules such as proteins [36,37]. Here, we adopt the same protocols when modeling the 3D structure of a chromosome.

The number of lattice sites or volume of the cubic lattice is:

Eq. (1) $$V=s^{3}$$,

where s is *s* = 5*n* with *n* as the number of beads. This bigger space (compared to setting each side of the cubic lattice to be n) allows enough free space to simulate the 3D structure.

We used a cooling schedule based on [38], in which we set the starting temperature *T*_0_ = 10. The decrement of temperature is:

Eq. (2) $$T_{c}=0.9^{c}*T_{0}$$,

where c is the number of times that temperature has been decremented, and *T_c_* is the current temperature. In this way, the temperature will keep decreasing with a rate of 0.9 every time the temperature’s value is updated. At the beginning of the simulation process, the value of temperature is relatively high that makes the algorithm to have higher probability to accept non-optimal moves, that is, the moves that do not reduce the value of the loss function. In this way, the algorithm allows relatively larger alternations of the 3D structure exploring bigger conformational space. This is an important feature for the simulated annealing protocol because in this way the algorithm may jump out of local minima. Towards the later stages of the simulation process, the value of temperature will be gradually reduced, which will make it stricter and stricter at accepting non-optimal moves. In this way, the algorithm will be more and more constringent, which means it will only allow small refinements of the 3D structure when the simulation is reaching the later stages. The following two equations will further explain this mechanism.

We repeated trials at each temperature until the system stabilized at that temperature. At each temperature, if on average there are 10 accepted moves per DNAbead or the number of trials exceeds 100 times of the number of beads, the algorithm will decrease the temperature based on Eq. (2) and continue running with the new temperature. If the desired acceptance number, that is, on average 10 accepted moves per bead, is not achieved for three consecutive temperatures, the annealing process is stopped.

For each trial, the algorithm randomly selects a DNA bead to move and then accepts the move with probability:

Eq. (3) $$P=e^{-\Delta E/T}$$

if Δ*E* ≥ 0 (Δ*E* ≥ 0 means it is not an optimal move, i.e., it is the move makes the 3D structure to have a bigger discrepancies with the target distances between all bead pairs), or always accepts the move if Δ*E* < 0 (Δ*E* < 0 means the move reduced the value of loss function, i.e., making the new 3D structure to better fit the target distances between all bead pairs), where T is the current temperature and Δ*E* is the change of the loss value after the move:

Eq. (4) ΔE=L(new)−L(old)

The loss function we used is:

Eq. (5) $$L=\sum_{i,j}^{n}w*{\left(d_{ij}-\delta_{ij}^{\prime }\right)}^{2}/{\delta_{ij}^{\prime }}^{2}$$,

where *w* is a parameter to scale the value of the loss value (for this study *w* = 0.0001), *d_ij_* is the actual Euclidean distance between beads *i* and *j* in the current structure, and $\delta_{ij}^{\prime }$ is the normalized target distance between beads *i* and *j*. The square of the target distance ${\delta_{ij}^{\prime }}^{2}$ is also put as the denominator because doing that will better normalize extremely large or small target values. The $\delta_{ij}^{\prime }$ is re-scaled from *δ_ij_* that is:

Eq. (6) $$\delta_{ij}=c_{ij}^{-1/3},c_{ij}\neq 0$$,

where *c_ij_* is the number of normalized Hi-C contacts between beads *i* and *j*. Eq. (6) including the parameter −1/3 is a classic approach to convert the number of Hi-C contacts to Euclidean distances in the 3D space that is motivated by polymer physics. Eq. (6) has been widely used by existing Hi-C-based 3D modeling methods [11,16], in which higher Hi-C values result in smaller Euclidean target distances. We normalized the value of the target distance by using:

Eq. (7) $$\delta_{ij}^{\prime }=n\sqrt{3}\frac{\delta_{ij}}{\max\left(\delta_{ij}\right)}$$,

where $n\sqrt{3}$ is the maximum allowed target distance in the 3D structure, that is also the diagonal length of a cubic lattice whose side length is the number of beads n, and *max* (*δ_ij_*) is the maximum target distance calculated from Hi-C contacts. This normalization process makes sure that the normalized target distance will fit within the size of our cubic lattice.

The population Hi-C contact matrices may contain many zero values due to reasons such as experimental imperfection, especially at high resolution. These zero values cannot be converted to target distances by Eq. (6) (would be infinity). Therefore, we use a 2D Gaussian function Eq. (8) to impute the zero values in the Hi-C contact matrices. This mechanism is based on 2D sequential proximity between DNAbead pairs. Specifically, if one bead pair on the DNAhas lots of Hi-C contacts, its sequentially adjacent bead pairs may not have zero Hi-C contact. In other words, the number of Hi-C contacts for a zero-Hi-C bead pair can be modeled based on the Hi-C contact values of its adjacent bead pairs. Specifically, it can be modeled as:

Eq. (8) $${c^{\prime }}_{ij}=\sum_{\left|a-i\right|\leq d_{0}\;\text{and}\;\left|b-j\right|\leq d_{0}}^{c_{ij}=0,c_{ab}>0}\frac{\left(-\left(\frac{{\left(a-i\right)}^{2}}{\mu }+\frac{{\left(b-j\right)}^{2}}{\mu }\right)\right)}{n^{\prime }}$$

In Eq. (8), *c_ij_* indicates the original zero Hi-C value between beads *i* and *j*; ${c^{\prime }}_{ij}$ indicates the imputed Hi-C value between beads *i* and *j*. The non-zero values of normalized Hi-C contact between beads *a* and *b* are used to impute the value between beads *i* and *j*. The value *n*′ indicates the number of neighboring bead pairs whose non-zero Hi-C values are used to impute the value for beads *i* and *j*. The *a* and *b* values are the X and Y coordinates in the 2D Hi-C contact matrix, which are in the range of (i-*d*_0_, i+*d*_0_) and (j-*d*_0_, j+*d*_0_). The value *d*_0_ is a distance threshold that controls the amount of surrounding non-zero values that will be used for the imputation. The parameter *μ* controls how much influence a non-zero value can generate to the originally zero value. A larger *μ* results in smaller influence. The default values of *μ* and *d*_0_ are determined by the resolutions of the Hi-C data and the percentage of zero Hi-C values. The values of these parameters can be freely changed when executing our source code.

### 2.4 Generating high-resolution 3D structures

To generate a 3D structure at a high-resolution, for example, 40 kb resolution, our algorithm at first generates the 3D structure at 400 kb resolution using the protocol defined above and then adds nine beads in between every two consecutive 400 kb beads. The resulting resolution of the 3D structure will be 40 kb. The procedure of adding beads is the same as the way of initializing the 3D structure in the cubic lattice as previously mentioned.

After beads are added, the system starts new simulations on the high-resolution structure. The initial temperature for the simulations is set to 0.1, and the number of trials at each temperature is five times of the number of the beads at 40 kb resolution. The reason why we used fixed temperature and trials here is that these simulations are performed on the 400 kb structure that has already been optimized. Therefore, we would only need to refine the structure instead of largely altering it. This will also help ensure to be able to generate high-resolution structures within reasonable amount of time.

### 2.5 3D structure of the X-chromosome during XCI

The distance of each bead pair traveling towards each other or further away from each other from 0 hours (before XCI) to 48 hours (after XCI) should equal the sum of travelled distances at each time interval, that is, from 0 hours to 3 hours, 3 hours to 6 hours, 6 hours to 24 hours, and 24 hours to 48 hours. This is modeled by the equation:

Eq. (9) $$v_{0h\_ij}\left(3-0\right)+\left(\frac{R_{3h\_ij}}{R_{0h\_ij}}\right)v_{0h\_ij}\left(6-3\right)+\left(\frac{R_{6h\_ij}}{R_{3h\_ij}}\right)v_{3h\_ij}\left(24-6\right)+\left(\frac{R_{24h\_ij}}{R_{6h\_ij}}\right)v_{6h\_ij}\left(48-24\right)=\delta_{48h\_ij}-\delta_{0h\_ij}$$

In Equation (9), *v*_0*h_ij*_ is the relative average traveling speed between beads *i* and *j* during the 0 hours to 3 hours period; (3 – 0) is the traveling time for beads *i* and *j* from 0 hours to 3 hours with speed *v*_0*h_ij*_. We used *v*_0*h_ij*_(3 – 0) to model the distance the beads *i* and *j* have traveled towards or further away from each other from 0 hours to 3 hours after XCI starts. Similarity, $\left(\frac{R_{3h\_ij}}{R_{0h\_ij}}\right)$ *v*_0*h_ij*_(6 – 3) is the distance beads *i* and *j* have traveled from the 3 hours to 6 hours after XCI starts, in which:

Eq. (10) $$R_{0h\_ij}=\left(RAP_{0h\_i}+RAP_{oh\_j}\right)/2$$,

where *RAP*_0*h_i*_ and *RAP*_0*h_j*_ are the RAP values on beads *i* and *j* respectively at 0 hours that were used to indicate the intensity of Xist localization on DNA. Therefore, the average of these two values, which is the *R*_0*h_ij*_ value in Eq. (10), is the Xist localization intensity of the beads *i* and *j*. The *R*_3*h_ij*_, *R*_6*h_ij*_, and *R*_24*h_ij*_ in Eq. (9) are calculated using the same way.

At first, we used constant speed and assumed no acceleration for bead pairs’ traveling (we would later model it with acceleration considered). We further assume that higher average RAP value on two beads will result in higher speed for the two beads’ traveling:

Eq. (11) $$v_{3h\_ij}=\left(\frac{R_{3h\_ij}}{R_{0h\_ij}}\right)v_{0h\_ij}$$,

where *v*_3*h_ij*_ is the constant speed from 3 hours to 6 hours after XCI starts. This assumption is based on the study in [34] that proposes a model for how Xist exploits and alters 3D genome structure to spread across the X-chromosome. Specifically, Engreitz *et al.* [34] proposed that Xist exploits the existing 3D structure of the X-chromosome to search for target sites to localize. After encountering a new site, Xist transcripts bind to that region and accumulate at spatially proximal sites of active gene-dense regions. By silencing the active region into Xist silenced compartment, Xist effectively pulls new regions of active chromatin closer to the Xist transcription locus, which eventually changes the 3D conformation of the chromosome.

Similarity, we set $\left(\frac{R_{6h\_ij}}{R_{3h\_ij}}\right)$ as the ratio between *v*_3*h_ij*_ and *v*_6*h_ij*_:

Eq. (12) $$v_{6h\_ij}=\left(\frac{R_{6h\_ij}}{R_{3h\_ij}}\right)v_{3h\_ij}$$,

where *v*_6*h_ij*_ is the constant speed from 6 hours to 24 hours after XCI starts. In Eq. (9), $\left(\frac{R_{6h\_ij}}{R_{3h\_ij}}\right)v_{3h\_ij}\left(24-6\right)$ is the travelled distance between beads *i* and *j* from 6 to 24 hours after XCI starts. We used the same method to calculate the distance beads *i* and *j* have travelled between 24 hours and the end of inactivation (48 hours).

The right of the equal sign in Eq. (9) indicates the total travelled distance from the beginning of XCI (0 hours) to the end of XCI (48 hours), in which *δ*_0*h_ij*_ and *δ*_48*h_ij*_ are the target distances between beads beads *i* and *j* at 0 hours and 48 hours that are converted from the number of normalized Hi-C contacts. From Eq. (9), we can solve the equation and get the value of *v*_0*h_ij*_. After that, we can calculate *v*_3*h_ij*_, *v*_6*h_ij*_, and *v*_24*h_ij*_, i.e., the speeds from 3 hours to 6 hours, 6 hours to 24 hours, and 24 to 48 hours. This allows us to further calculate the target distance between each bead pair at 3 hours, 6 hours, and 24 hours since we know the target distance at 0 hours and the speed and traveled time during each period of time.

**Figure 1.**
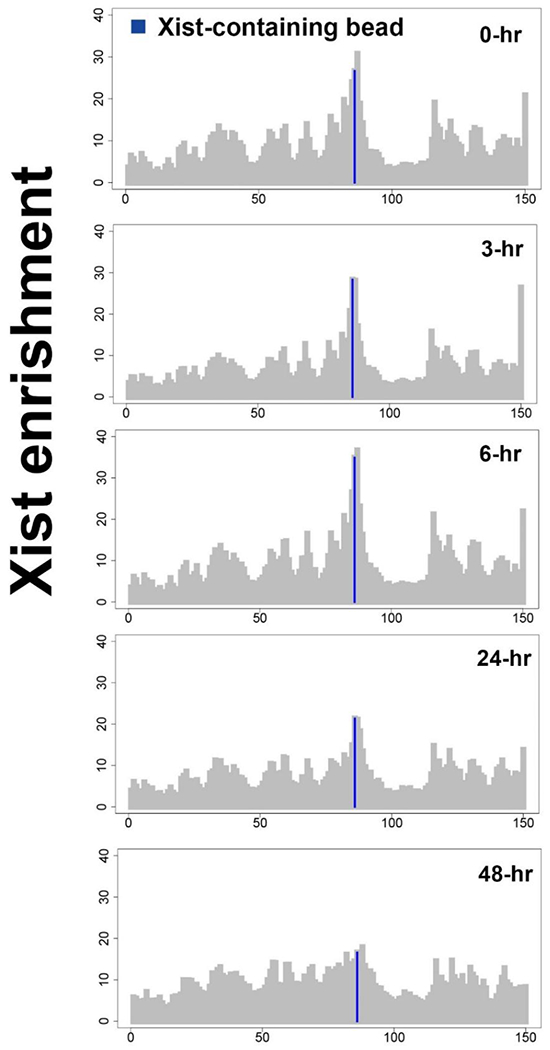
The Xist localization intensities at different time points during the XCI process (0 hours, 3 hours, 6 hours, 24 hours, and 48 hours after XCI starts).

Figure 1 shows the Xist localization intensity (RAP data) on the X-chromosome 0 hours, 3 hours, 6 hours, 24 hours, and 48 hours after XCI starts. It can be found that at the beginning of XCI, e.g., between 0 hours and 3 hours, the Xist locus has the highest level of Xist RNA, followed by the regions that are spatially proximate to Xist locus. We assume this high intensity of Xist localization results in higher speed of Xist pulling the proximate region towards Xist locus. In comparison, the 5’ region of the X-chromosome tends to have less localized Xist RNA. Therefore, the 3D conformation at that region is not intensively altered, corresponding to smaller traveling speed at that specific region.

At 24 and 48 hours after XCI, the localization intensities of Xist transcripts in the Xist locus dropped (see Figure 1), similar to some other spatially proximal regions. In that case, our model assigns reduced traveling speed for the bead pairs in those regions. This makes sense as the 3D conformation in those regions have already been altered at the beginning of XCI. Therefore, the intensity of the 3D conformation being changed is reduced, which indicates smaller traveling speed at the later stage of XCI.

We also model the travelling of bead pairs considering a different acceleration value for every periods of time. Similarly as in Eq. (9), the distance of each bead pair that had travelled towards each other or further away from each other from 0 hours (before XCI) to 48 hours (after XCI) after XCI starts should equal the sum of travelled distances at each time period, that is, from 0 hours to 3 hours, 3 hours to 6 hours, 6 hours to 24 hours, and 24 hours to 48 hours. This is modeled by the equation:

Eq. (13) $$\left[v_{0h\_ij}.\left(3-0\right)+\frac{1}{2}.a_{0h\_ij}{\left(3-0\right)}^{2}\right]+\left[v_{3h\_ij}.\left(6-3\right)+\frac{1}{2}.a_{3h\_ij}.{\left(6-3\right)}^{2}\right]+\left[v_{6h\_ij}.\left(24-6\right)+\frac{1}{2}.a_{6h\_ij}.{\left(24-6\right)}^{2}\right]+\left[v_{24h\_ij}.\left(48-24\right)+\frac{1}{2}.a_{24h\_ij}.{\left(48-24\right)}^{2}\right]=\delta_{48h\_ij}-\delta_{0h\_ij}$$,

where *v*_0*h_ij*_ is the initial velocity of bead *i* and bead *j* at 0 hours; and we assumed it as zero. The travelling time for the first time period is (3 – 0) hours. The *a*_0*h_ij*_ is the acceleration of bead *i* and bead *j* during 0 to 3 hours.

The first two terms in Eq. (13): $v_{0h\_ij}.\left(3-0\right)+\frac{1}{2}.a_{0h\_ij}.{\left(3-0\right)}^{2}$ represent the travelled distance of bead *i* and bead *j* during 0 to 3 hours after XCI starts. Similarity, *v*_3*h_ij*_, *v*_6*h_ij*_, and *v*_24*h_ij*_ are the initial velocities of bead *i* and bead *j* for three time periods: 3 to 6 hours, 6 to 24 hours, and 24 to 48 hours. The *a*_3*h_ij*_, *a*_6*h_ij*_, and *a*_24*h_ij*_ are the accelerations for bead *i* and bead *j* in different time periods respectively.

As Xist transcripts cause the structural change of the X-chromosome, we model the acceleration with respect to the localization intensity of Xist transcripts or the RAP values as:

Eq. (14) $$a_{3h\_ij}=\frac{R_{3h\_ij}}{R_{0h\_ij}}a_{0h\_ij}$$,

where *R*_0*h_ij*_ is the average Xist localization intensity of bead *i* and bead *j* at 0 hours calculated as:

Eq. (15) $$R_{0h\_ij}=\frac{1}{2}.\left(RAP_{i\_0h}+RAP_{j\_0h}\right)$$,

where parameters *RAP*_*i*_0*h*_ and *RAP*_*j*_0*h*_ are the Xist localization intensities for bead *i* and bead *j* at 0 hours respectively.

From Eq. (13), the value of *a*_0*h_ij*_ can be calculated. After that, we can get all the acceleration values for all time periods. Thus, the distances of bead *i* and bead *j* approaching or moving away from each other in each XCI time period can be calculated, and we can further obtain the target distance between every bead pair at 3 hours, 6 hours, and 24 hours after XCI starts.

## 3. Results

### 3.1 Low-resolution 3D structures of the active and inactive X-chromosomes for neural progenitor cells and embryonic stem cells

**Figure 2.**
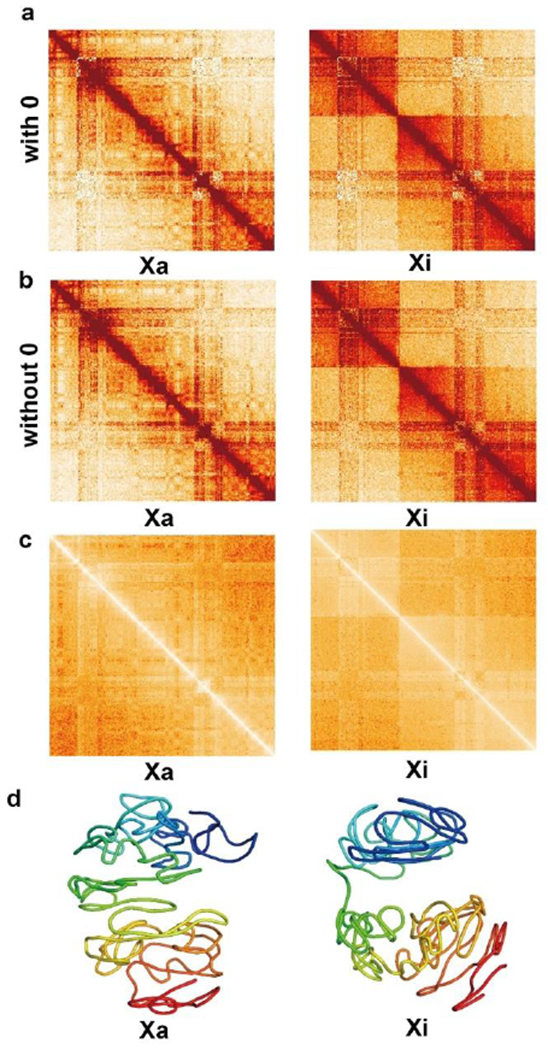
(a) 1 Mb resolution heatmaps of the original Hi-C contacts of the NPC X-chromosome (both active and inactive X-chromosomes). Zero values are included. (b) The heatmaps of the Hi-C contact matrices of the NPC X-chromosome, in which the zero values have been imputed by the 2D Gaussian function. (c) The heatmaps of target distances between all bead pairs of the active and inactive X-chromosomes of NPC. (d) 1 Mb resolution structures of the active (0 hours) and inactive (48 hours after XCI starts) X-chromosomes of NPC.

We resized the Hi-C data downloaded from [35] at 500 kb resolution of the active and inactive NPC X-chromosomes into 1 Mb low resolution. Figure 2 (a) shows the 1 Mb resolution Hi-C contact heatmaps of the X-chromosome of NPC. These heatmaps indicate the normalized Hi-C data, in which the “none” entries have been deleted with darker color indicating higher Hi-C values. The Hi-C matrices in Figure 2 (a) contains zero values.

Figure 2 (b) shows the heatmaps of Hi-C contacts for the NPC X-chromosome, in which the “none” entries have been removed and the zero values have been imputed by the 2D Gaussian function. Therefore, there are no zero values in the Hi-C matrices; all of the values in the Hi-C contact maps can be converted to target distances between bead pairs. Target distances are the Euclidean distances between bead pairs in the 3D space that our method uses as the target to achieve. In other words, our method tries to make the real distances between all bead pairs as close as possible to their target distances.

Figure 2 (c) shows the heatmaps of the target distances. We used these target-distance matrices as the input when modeling the 3D structures of the X-chromosome. The 3D structures were generated using the algorithms discussed in the Methods section. The structures of the active (0 hours) and inactive X-chromosomes (48 hours after Xist expression) of NPC at 1 Mb resolution are shown in Figure 2 (d).

The results for the mouse ES cells can be found in Supplementary Figure S1.

### 3.2 Validation of the lattice-based approach for generating 3D chromosome structures

Supplementary Figure S2 shows the values of the loss function from the first trial to the last trial during the simulation process. It can be seen that our simulated annealing algorithm was able to keep reducing the loss function.

To validate the accuracy of our lattice-based model, we compared the 3D structure our method generated with the one constructed by PASTIS. We executed PASTIS using the same loss function as we used in Eq. (5) with the difference that PASTIS uses the software IPOPT [16] to solve the optimization problem.

We constructed the 1 Mb resolution 3D structure of the active X-chromosome (Xa) that was derived from neural progenitor cells (NPC) of hybrid mice [35]. In total, 40 jobs were executed independently to 40 3D structures in total. In this way, a pool of 40 structures, an ensemble, was generated. After that, we used Q-score [39] to select the top three structures. The Q-score of a target structure is the average of the pair-wise comparison, that is, the TM-score [40], between the target structure and all other structures in the pool (in this case, other 39 structures). TM-score [40] is an algorithm for measuring the structural similarity between two protein 3D structures, in which zero indicates no similarity between the two structures and one indicates that the two structures are exactly the same. It has been proven in the protein structure prediction community that the top predicted structure(s) ranked by the Q-score (also called clustering method) usually best match the native structure [41]. Therefore, Q-score has been largely used to pick up the best predicted structure from a pool of predicted protein structures. Here, we also used Q-score to pick up the most representative top three structures and then compare them with the structure generated by PASTIS.

**Figure 3.**
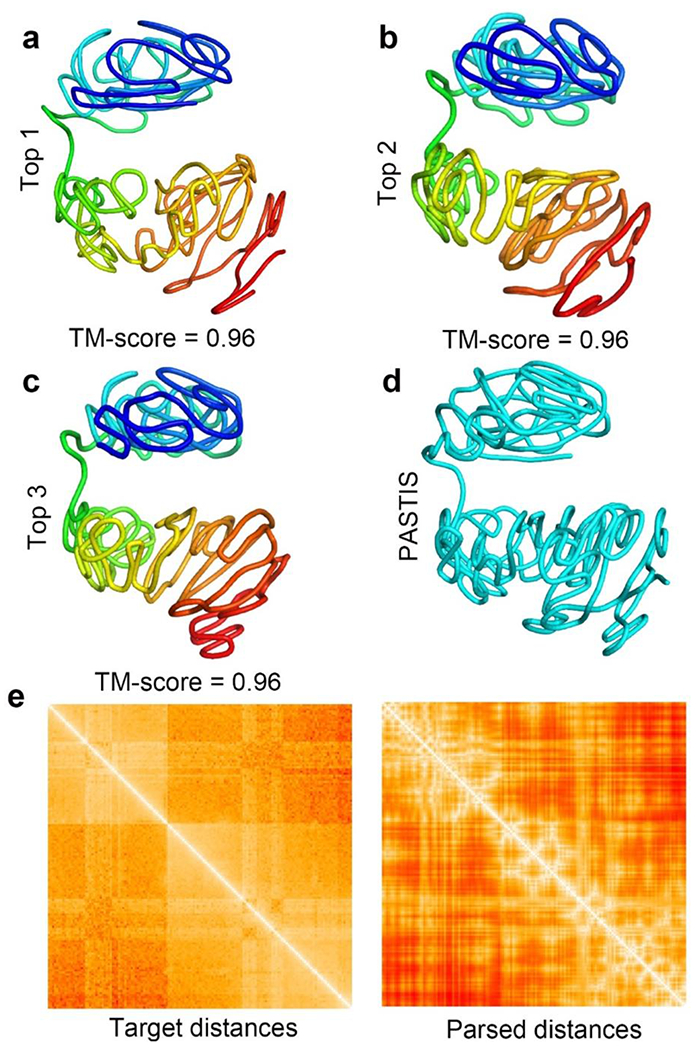
(a)–(c) The top three 1 Mb structures inactive NPC X-chromosome generated by our modeling method. The TM-score between the structure generated by PASTIS and the top three structures our method generated are listed in the figure. (d) The 1 Mb resolution 3D structure of the inactive NPC X-chromosome that was generated by PASTIS. (e) (left) The heatmap of target distances between all bead pairs of the inactive X-chromosome and (right) the Euclidean distances parsed from the 3D structure our approach reconstructed.

Figure 3 (a)–(c) show our top three structures of the inactive X-chromosome at 1 Mb resolution ranked by Q-score. Figure 3 (d) shows the 3D structure constructed by PASTIS. The TM-scores between the top three structures and PASTIS structure are all 0.96. Besides using the top-three structures, we also compared all of the 40 structures with the PASTIS structure. The lowest TM-score between our 40 structures and PASTIS structure is 0.93 and the highest is 0.98 (the distribution of the 40 TM-scores is plotted in Supplementary Figure S3). These high TM-scores indicate that our lattice-based method was correctly implemented, and our approach can generate stable structures.

Figure 3 (e) shows the heatmap of the target distances for the inactive X-chromosome (48 hours) and the heatmap of the Euclidean distances parsed from the top-one 3D structure we reconstructed. The Pearson’s correlation between the two heatmaps (matrices) is 0.93 with a p-value < 0.00001 indicating that our 3D modeling method successfully reconstructed the 3D structure, i.e., made a high correlation between target and actual distances in the reconstructed 3D structure. The correlations and p-values at different stages of the XCI process are shown in Supplementary Figure S4 (1 Mb resolution) and Supplementary Figure S5 (250 kb resolution).

### 3.3 3D structure of the NPC X-chromosome during the process of X-chromosome inactivation

**Figure 4.**
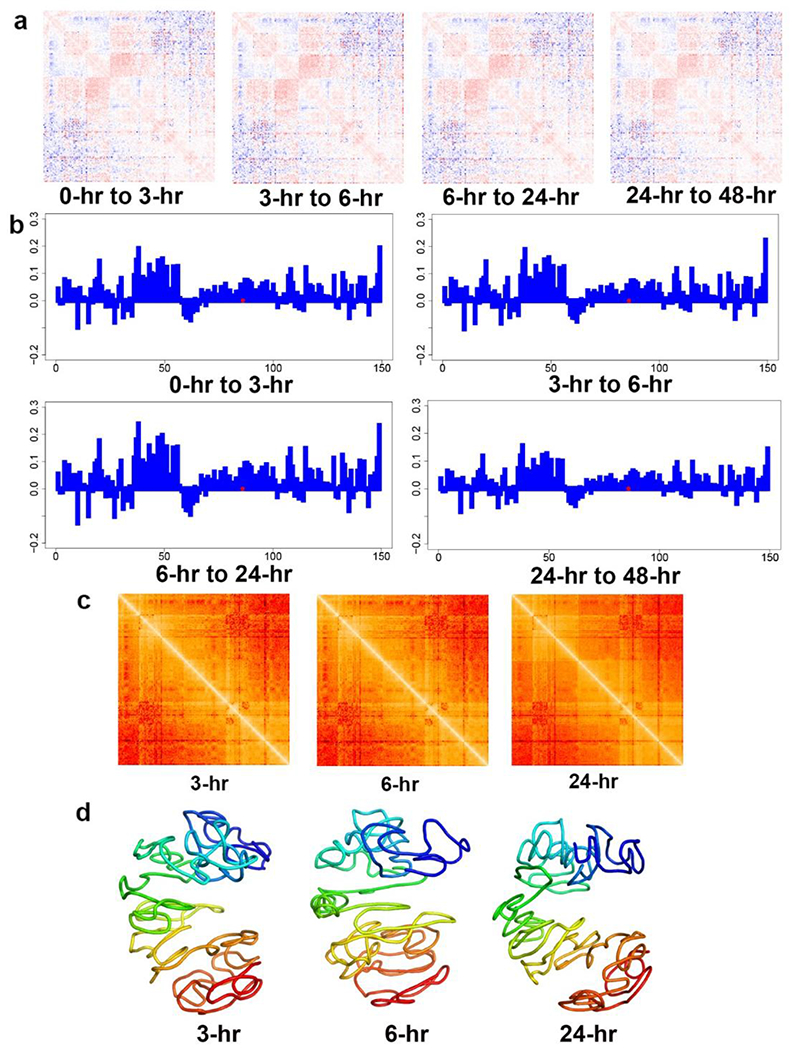
(a) The heatmaps of the traveling speeds between all bead pairs during different time periods of XCI. The red and blue colors in the heatmaps indicate the positive (getting closer) and negative (away from each other) traveling speeds of every bead pair, respectively. (b) Shown at 1 Mb resolution: The traveling speeds between Xist-containing bead and other beads at different time periods of XCI for the NPC X-chromosome. The dotted red line indicates the location of the Xist locus. (c) The heatmaps of target distances between all bead pairs of the NPC X-chromosome at 3 hours, 6 hours, and 24 hours after XCI starts at 1 Mb resolution. (d) 1 Mb resolution 3D structures of the NPC X-chromosome at 3 hours, 6 hours, and 24 hours after XCI starts.

Figure 4 (a) shows the travelling speed (assuming no acceleration) of each bead pair at different time periods during XCT The travelling speeds between the bead that contains Xist locus and all other beads are indicated in Figure 4 (b), in which the X-axis represents every bead in the X-chromosome and the Y-axis represents the speed.

Based on the speed values shown in Figure 4 (a), we calculated the target distances at 3 hours, 6 hours, and 24 hours after XCI starts as shown in Figure 4 (c). Based on the target distances, we modeled the 1 Mb resolution structures at 3 hours, 6 hours, and 24 hours after XCI starts, as shown in Figure 4 (d).

**Figure 5.**
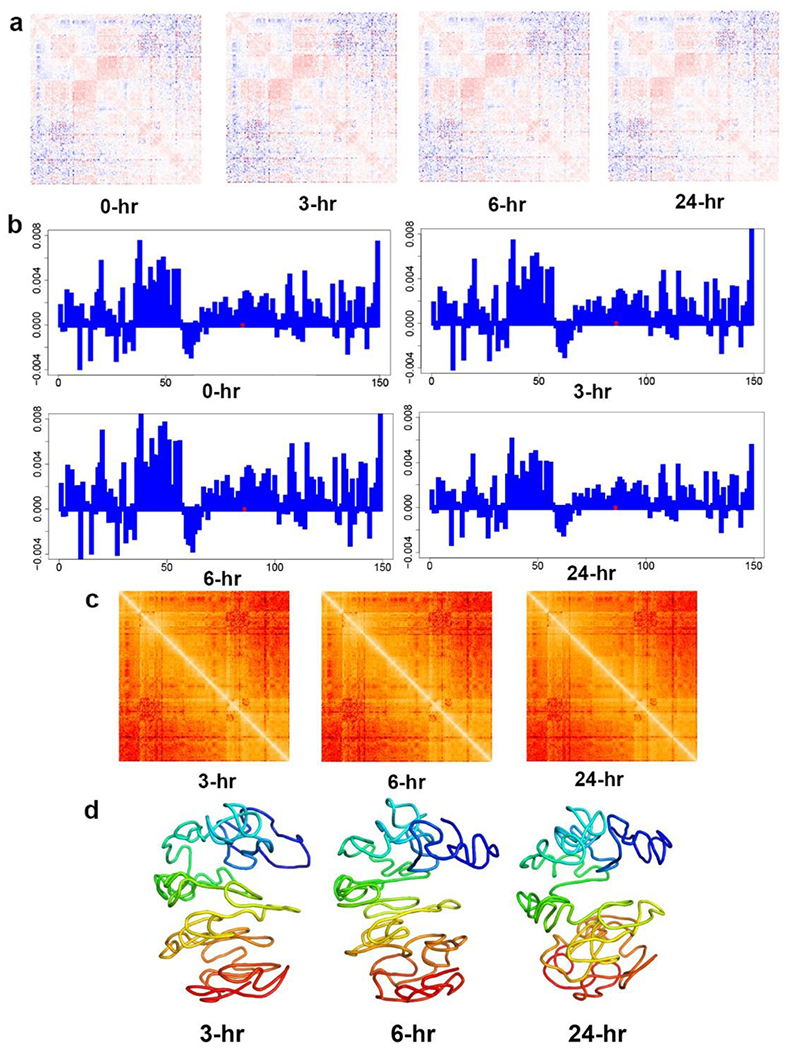
(a) The heatmaps of the acceleration values between all the bead pairs in the NPC X-chromosome at different time periods. The red and blue colors in the heatmaps indicate the positive (getting closer) and negative (away from each other) accelerations of every bead pair, respectively. (b) Shown at 1 Mb resolution: The accelerations between Xist locus containing bead and all other beads in the X-chromosome at four XCI time periods. (c) The heatmaps of the target distances at three time points, 3 hours, 6 hours, and 24 hours after XCI starts (with acceleration considered). (d) The 3D structures of the X-chromosome at different time points according to the target distances shown in (c).

Figure 5 shows the similar contents when acceleration is considered. Specifically, Figure 5 (a) shows the heatmaps of acceleration values at different time periods of XCI. Figure 5 (b) shows the acceleration between the Xist-locus-containing bead and all other beads. Figure 5 (c) indicates the target distances at three time points during the XCI (3 hours, 6 hours, and 24 hours after XCI starts).

### 3.4. High-resolution 3D structures of the NPC X-chromosome during the process of XCI

**Figure 6.**
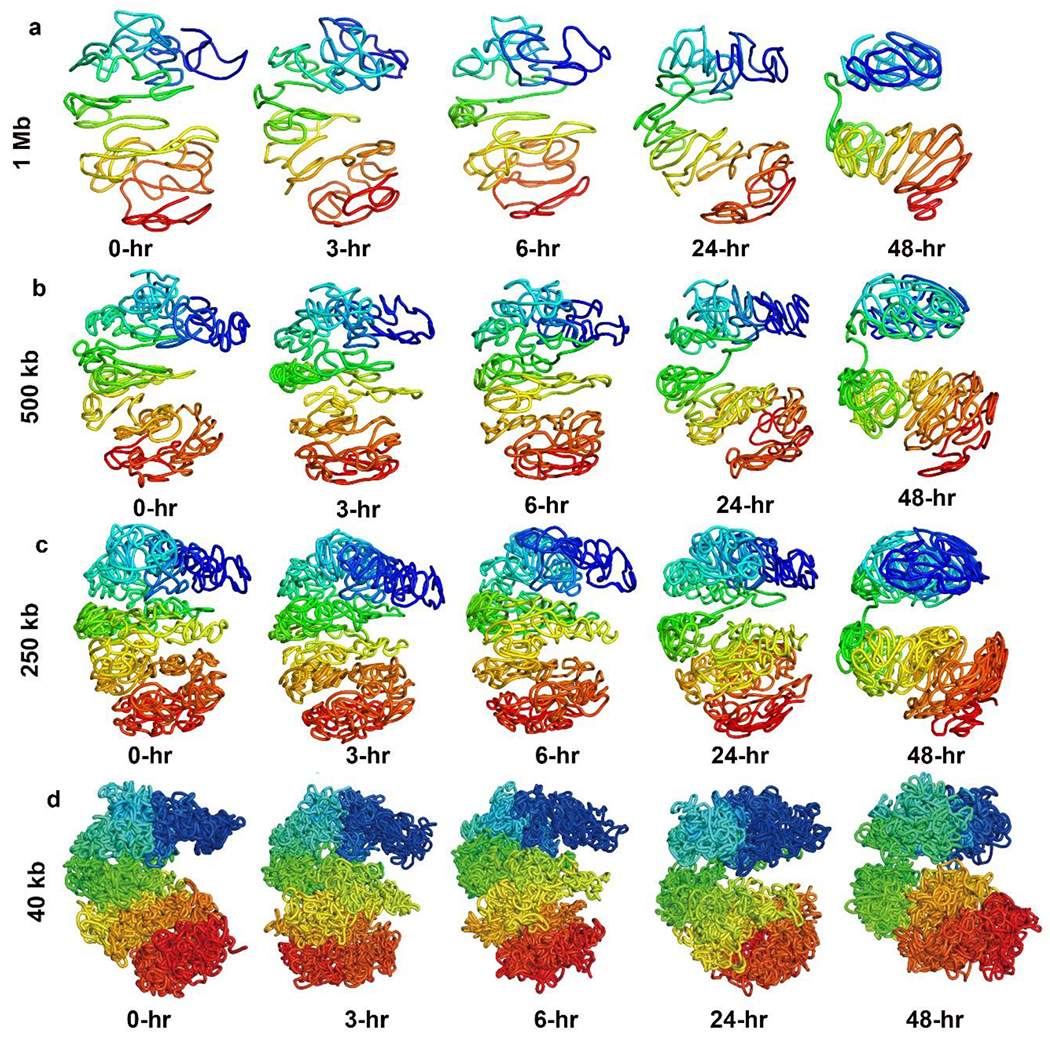
(a)–(d) The reconstructed 3D structures of the NPC X-chromosome at 0 hours (before XCI), 3, 6, and 24 hours (during XCI), and 48 hours (after XCI); at 1 Mb, 500 kb, 250 kb, and 40 kb resolutions. Constant traveling velocity is used to model the travelled distances.

**Figure 7.**
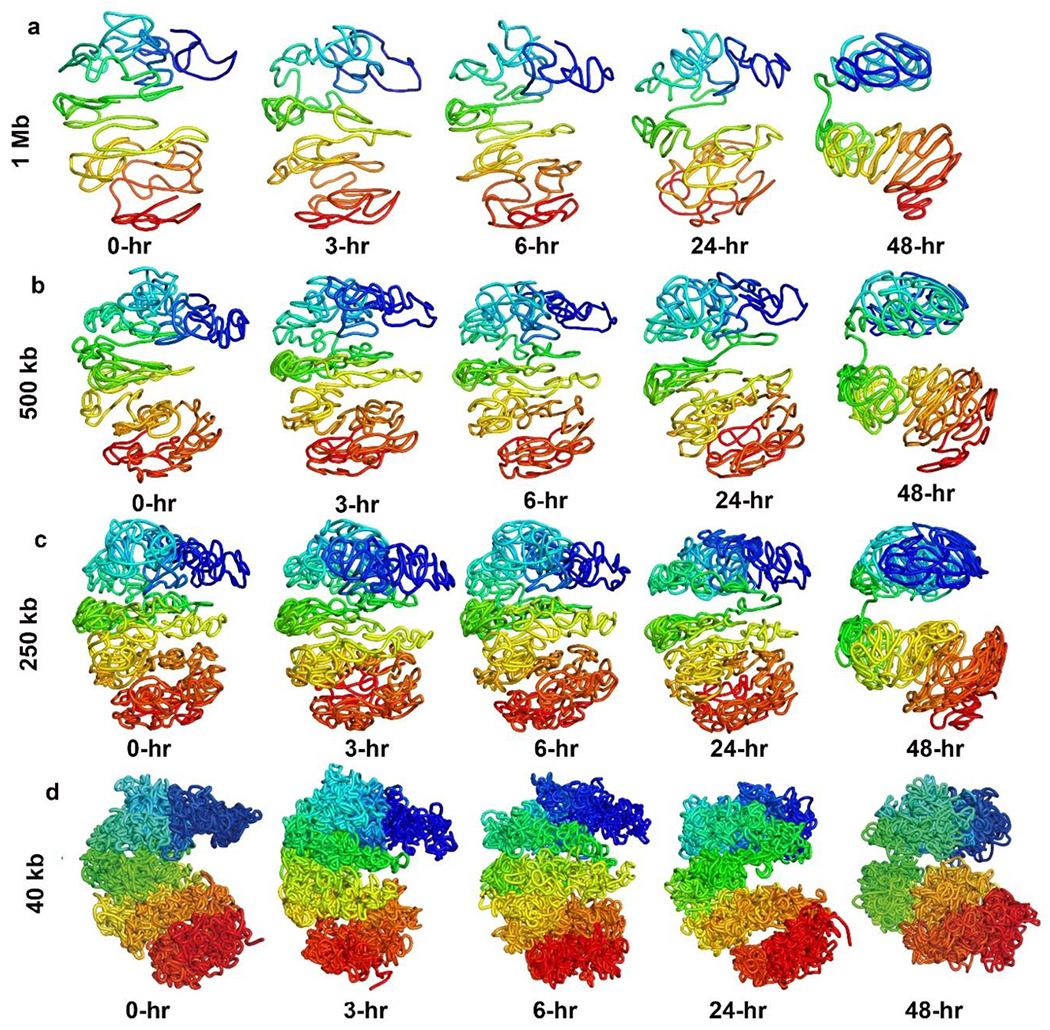
(a)–(d) The reconstructed 3D structures of the NPC X-chromosome at 0 hours (before XCI), 3, 6, and 24 hours (during XCI), and 48 hours (after XCI) at 1 Mb, 500 kb, 250 kb, and 40 kb resolutions. Acceleration was considered when modeling the travelled distances.

Our lattice-based model can also generate high-resolution 3D structures of the X-chromosome. Figure 6 (a)–(d) show the 3D structures we reconstructed (when assuming constant travelling speed) for the NPC X-chromosome at 0 (before XCI starts), 3, 6, and 24 hours (during XCI), and 48 hours (after XCI) at 1 Mb, 500 kb, 250 kb, and 40 kb resolutions. It clearly shows how the 3D structure of the X-chromosome gradually changed into two large domains, also known as a bipartite structure. Figure 7 shows the structures when acceleration is considered.

## 4. Discussion

We developed a new approach to model the chromosome 3D structure based on population Hi-C data. Our approach is based on simulations performed on a 3D lattice. Furthermore, we used the method to reconstruct the 3D structures of the X-chromosome during the XCI process. We first modeled the X-chromosome in mouse NPC cells. From 40 independent simulations, we selected the top three structures based on the Q-score and then compared them with the structure generated by PASTIS. Each of the top three structures has a TM-score of 0.96. We then compared all of the 40 structures with PASTIS structure and found that all of them have a TM-score at around 0.96. This indicates that our approach has been correctly implemented and that the structures generated by our approach are stable. The Pearson’s correlation between the target distances converted from the Hi-C contact matrices and the Euclidean distances parsed from the reconstructed 3D structure at 1 Mb resolution is 0.93. It proves that our model can successfully generate the 3D structures.

We also proposed and implemented a 2D Gaussian method to model the zero values in the Hi-C contact maps, that is, making the zero values non-zero based on their sequential distances to the neighboring bead pairs that originally have non-zero Hi-C contacts. This approach can be largely used as an imputation procedure on the Hi-C contact maps.

We downloaded the Xist localization intensity data (RAP) data for NPC and ES cells at 0, 3, 6, 24, and 48 hours after XCI starts. Based on the previous finding that Xist spreads to spatially proximal site from the Xist locus and pulls the interacting segments to the Xist locus, we developed two methods to model the traveling speed and acceleration between every bead pair in the X-chromosome. The speed or acceleration of two beads traveling closer to or away from each other can be used to infer the target distances between all bead pairs at different time points in the XCI process.

Based on the traveling speed and acceleration, we further calculated the target distances between all bead pairs at different time points of XCI. Then, we reconstructed the 3D structures of the X-chromosome during XCI. These structures show how the active X-chromosome gradually changed into the inactive X-chromosome.

One may argue that the findings from [34] indicate that Xist pulls regions of active chromatin closer to the Xist transcription locus, but here our mathematical model assigns speeds not only for the bead pairs between the Xist-locus-containing bead and other beads, but also the bead pairs none of which contains Xist locus. We believe our mathematical model does not violate the previous finding that the regions of active chromatin are pulled towards the Xist locus. This is because the speed we assign to a bead pair that neither of which is Xist-locus-containing bead can be considered as the relative speed between the two beads even if both beads travel towards the Xist locus. Also, the speed in the middle of XCI process is based on the 3D structures before and after XCI (0 hours and 48 hours) that are inferred from Hi-C data. Therefore, if the spatial distance between a bead pair do change during the XCI process (even these two beads may both travel towards the Xist locus), it can be indicated by our mathematical model with a none zero speed (can be getting closer or apart). If the spatial distance between a bead pair did not change during XCI, their relative speed would be zero. Either way, it can be modeled by our mathematical model and fits the Hi-C (also the 3D structures inferred from Hi-C) before and after the XCI process.

## 5. Conclusion

Our 3D lattice-based approach can generate accurate and stable high-resolution 3D chromosome structures based on population Hi-C data. We used Xist localization intensity to infer the traveling speed and acceleration between DNAbead pairs during the process of XCI. We reconstructed the 3D structure of the X-chromosome at different time points of XCI. For the first time, this can show the changing of 3D structure of the X-chromosome during the XCI process.

## Supplementary Material

suppl
