# Supplementary material for: Inferring the three-dimensional structures of the X-chromosome during X-inactivation
Source: Math Biosci Eng. Author manuscript; Available in PMC 2020 Dec 30. (PMC7772933; doi:10.3934/mbe.2019369)
Supplement: suppl [file NIHMS1655905-supplement-suppl.pdf]

Supplementary document for

Inferring the three-dimensional structures of the X-chromosome during X-chromosome inactivation

Hao Zhu[1], Nan Wang[2], Jonathan Z. Sun[3], Ras B. Pandey[4], and Zheng Wang[1,*],

[1] Department of Computer Science, University of Miami, 1364 Memorial Drive, Coral Gables, FL 33124, USA

[2] Department of Computer Science, New Jersey City University, 2039 Kennedy Blvd, Jersey City, NJ 07305, USA

[3] Department of Computer Science, College of Charleston, Charleston, SC 29424, USA

[4] Department of Physics and Astronomy, University of Southern Mississippi, 118 College Drive #5046, Hattiesburg, MS 39406, USA

[*] Corresponding author, email zheng.wang@miami.edu

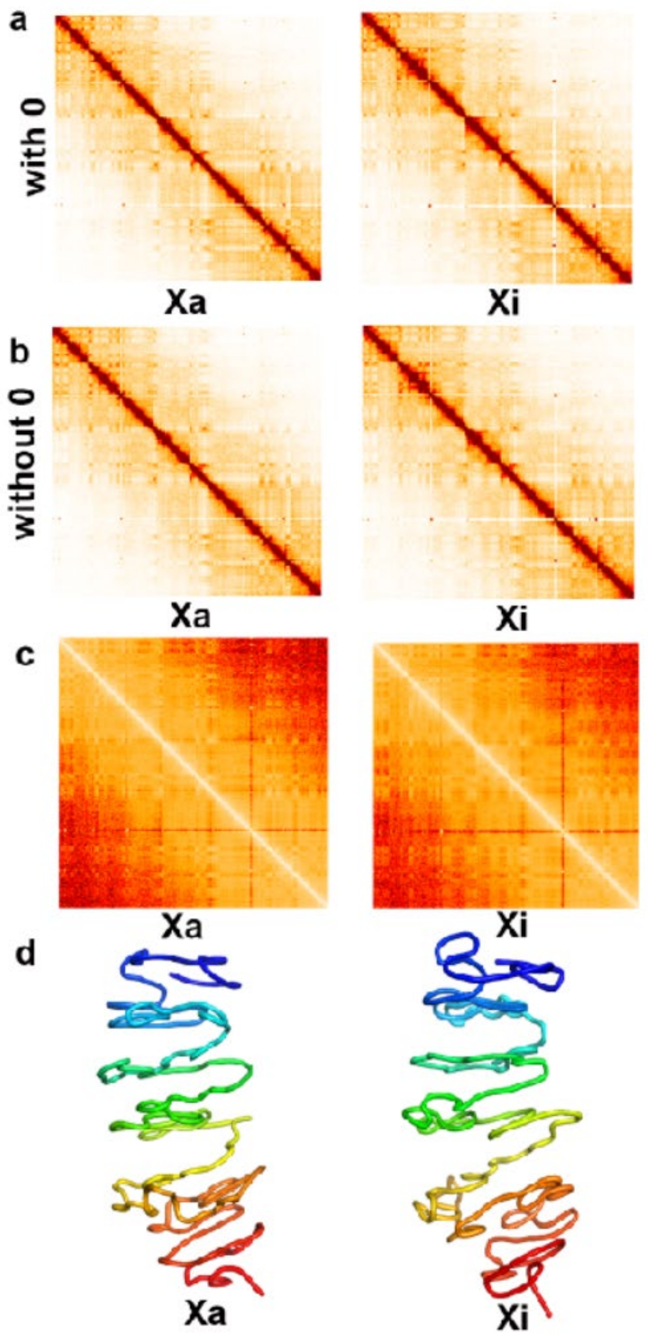

Figure S1. (a) The heatmaps of 1 Mb resolution original normalized Hi-C data for ES before the start of Xist expression (0 hour) and 48 hours after the start of Xist expression (X-chromosome inactivated). (b) the 1 Mb resolution heatmaps of the active and inactive ES Hi-C data, in which the "none" entries have been deleted and the zero values have been modeled by the 2D Gaussian function. (c) The heatmaps of target distances between all bead pairs of the X-chromosome 0 hour and 48 hours after the start of XCI for ES. (d) The structures of the active and inactive X-chromosome of ES cells at 1 Mb resolution.

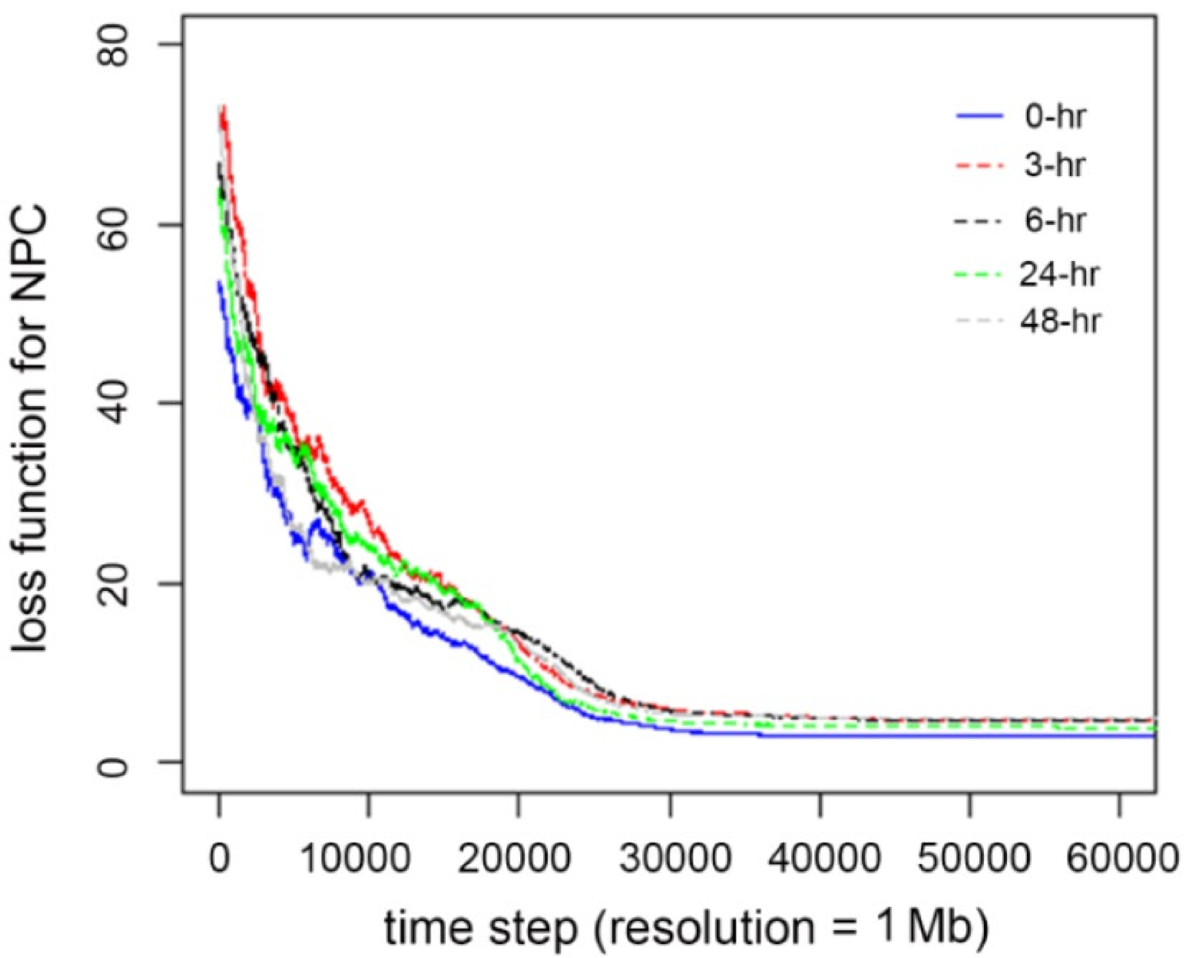

Figure S2. The values of the loss function from the first time-step to the last time-step when our simulated annealing stopped of NPC X-chromosome at different hours after X-chromosome inactivation started.

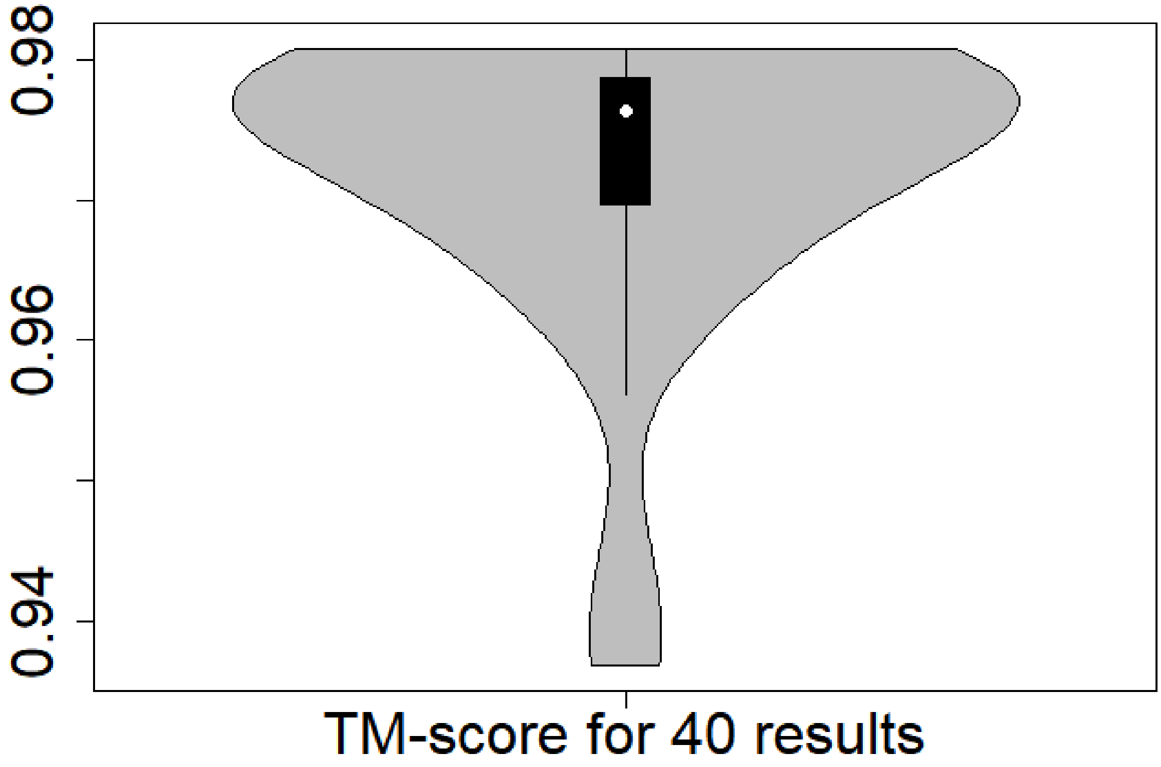

Figure S3. Distribution of the TM-scores between the 3D structure generated by PASTIS method and 40 3D structures our approach reconstructed.

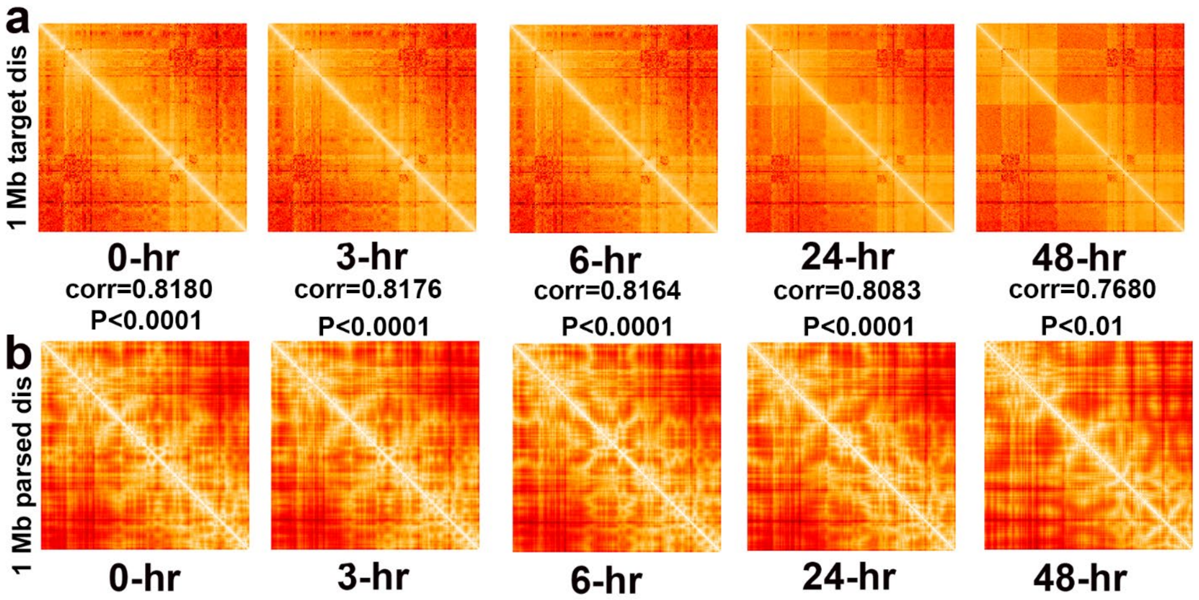

Figure S4. (a) 1 Mb resolution heatmaps of the target distances of NPC at five time points, 0, 3, 6, 24, and 48 hours after XCI starts. (b) Heatmaps of Euclidean distances parsed from the 3D structure our approach generated for the same time points as shown in (a). The correlations and the P-value between the two corresponding heatmaps are listed here.

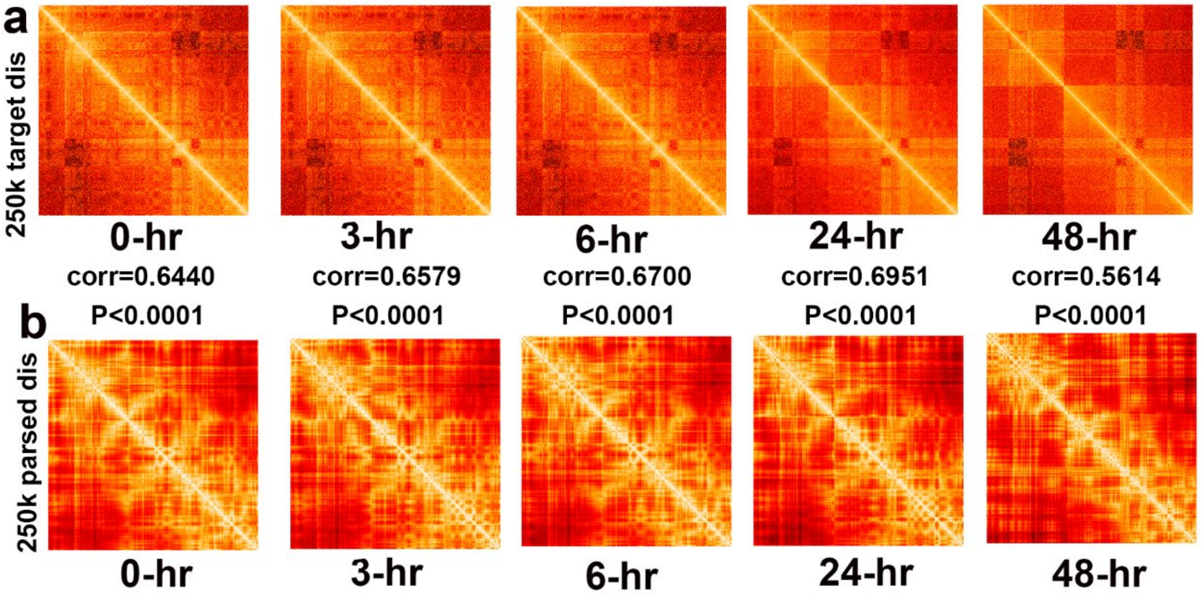

Figure S5. Figure S5. (a) 250 kb resolution heatmaps of the target distances of NPC at five time points, 0, 3, 6, 24, and 48 hours after XCI starts. (b) Heatmaps of Euclidean distances parsed from the 3D structure our approach generated for the same time points as shown in (a). The correlations and the P-value between the two corresponding heatmaps are listed here.
